# Promises of Plasmonic Antenna‐Reactor Systems in Gas‐Phase CO_2_ Photocatalysis

**DOI:** 10.1002/advs.202302568

**Published:** 2023-06-20

**Authors:** Zhijie Zhu, Rui Tang, Chaoran Li, Xingda An, Le He

**Affiliations:** ^1^ Institute of Functional Nano & Soft Materials (FUNSOM) Soochow University Suzhou 215123 P. R. China; ^2^ Jiangsu Key Laboratory for Carbon‐Based Functional Materials & Devices Soochow University Suzhou Jiangsu 215123 P. R. China; ^3^ Jiangsu Key Laboratory of Advanced Negative Carbon Technologies Soochow University Suzhou Jiangsu 215123 P. R. China

**Keywords:** antenna‐reactor, CO_2_ reduction, photocatalysis, photochemical effect, photothermal catalysis, plasmonic

## Abstract

Sunlight‐driven photocatalytic CO_2_ reduction provides intriguing opportunities for addressing the energy and environmental crises faced by humans. The rational combination of plasmonic antennas and active transition metal‐based catalysts, known as “antenna‐reactor” (AR) nanostructures, allows the simultaneous optimization of optical and catalytic performances of photocatalysts, and thus holds great promise for CO_2_ photocatalysis. Such design combines the favorable absorption, radiative, and photochemical properties of the plasmonic components with the great catalytic potentials and conductivities of the reactor components. In this review, recent developments of photocatalysts based on plasmonic AR systems for various gas‐phase CO_2_ reduction reactions with emphasis on the electronic structure of plasmonic and catalytic metals, plasmon‐driven catalytic pathways, and the role of AR complex in photocatalytic processes are summarized. Perspectives in terms of challenges and future research in this area are also highlighted.

## Graphical abstract

Plasmonic antenna‐reactor photocatalysts sustain numerous photophysical and photochemical mechanisms that facilitate CO_2_ photocatalysis, including resonant plasmonic energy transfer, hot carrier injection, photothermal effect, forced plasmons, and/or synergistic pathways.

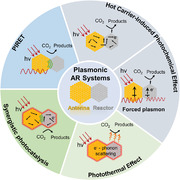

## 1 Introduction

The rapid consumption of fossil fuels has led to a sharp increase in the concentration of carbon dioxide (CO_2_) in the global atmosphere, resulting in severe consequences of environmental changes, such as global warming and ocean acidification.^[^ ^1^, ^2^, ^3^, ^4^ ^]^ Catalytic conversion of CO_2_ into value‐added products offers a promising strategy for mitigating the atmospheric CO_2_ concentration.^[^ ^5^, ^6^, ^7^, ^8^ ^]^ To overcome the energy barrier for CO_2_ activation, conventional metal‐based nanocatalysts typically operate under harsh conditions, leading to large fossil fuel consumption and byproduct generation.^[^ ^9^, ^10^, ^11^, ^12^, ^13^ ^]^ To address these challenges, photoexcitation has been utilized as an alternative energy source for CO_2_ activation and to provide selective control over CO_2_ catalysis.^[^ ^14^, ^15^, ^16^, ^17^, ^18^ ^]^

Photothermal catalysis represents one major pathway of CO_2_ photocatalysis, where sunlight is converted into thermal energy and drives the catalytic reactions.^[^ ^19^, ^20^, ^21^, ^22^, ^23^ ^]^ Since the pioneering study by Prof. Jinhua Ye and coworkers in 2014,^[^ ^24^ ^]^ many attempts have been made in the development of photothermal catalysts for CO_2_ conversion.^[^ ^25^, ^26^, ^27^, ^28^, ^29^, ^30^ ^]^ While they successfully achieved high conversion rates and catalytic efficiencies, photothermal catalytic pathways reported so far are primarily attributed to light‐assisted thermal catalytic mechanism, where the effect of light is limited to heat generation, and the catalysis is still thermal‐driven processes. Thus, the relatively high local temperature and the limited usage of light could still pose a series of challenges to catalytic stability and product selectivity.^[^ ^31^, ^32^, ^33^, ^34^ ^]^ A promising solution to these issues lies in the introduction of photochemical contributions.^[^ ^35^, ^36^, ^37^ ^]^ Photochemical effect functions through altering reaction pathways and reducing the activation barriers, ameliorating both the kinetics and thermodynamics of the catalytic reaction. Wide‐bandgap semiconductors are one common choice to introduce photochemical effects.^[^ ^38^, ^39^, ^40^, ^41^ ^]^ Yet their band structure and unfavorable charge recombination kinetics at elevated temperatures limits their photochemical conversion efficiency.^[^ ^42^, ^43^, ^44^, ^45^, ^46^, ^47^ ^]^ In more recent studies, plasmonic metals that possess large absorption cross‐sections in the visible region due to their signature localized surface plasmon resonance (LSPR) effect have emerged as one common choice for photochemical catalysis.^[^ ^48^, ^49^, ^50^, ^51^, ^52^, ^53^, ^54^, ^55^ ^]^ The excitation, dephasing, and relaxation of the surface plasmon can lead to a series of photophysical and photochemical phenomena, which provides plausible mechanisms for photochemical catalysis. However, despite their favorable optical properties, their *d* band generally centers far from the Fermi level, disfavoring the adsorption of reactants and intermediates, limiting their surface reactivity as efficient CO_2_ photocatalysts.

**Figure 1:**
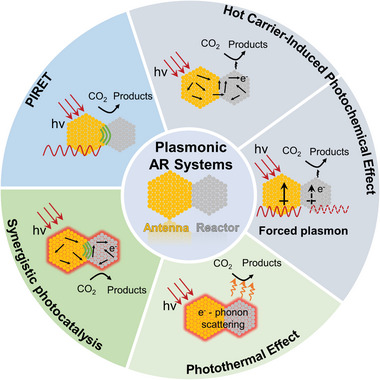
Mechanisms of CO_2_ photocatalysis over plasmonic AR systems.

The rational combination of plasmonic antennas and active transition metal‐based catalysts, known as “antenna‐reactor” (AR) nanostructures, allows the simultaneous optimization of optical and catalytic performances of photocatalysts, and thus holds great promise for CO_2_ photocatalysis. The concept of AR nanostructures was first proposed by Prof. Naomi J. Halas and coworkers in 2016.^[^ ^56^, ^57^ ^]^ Catalytic reactions could be facilitated through plasmon‐induced resonant energy transfer (PIRET), hot charge carrier‐mediated photochemistry, and/or photothermal effects (**Figure** **1**). Through modulation of the structure and composition of the antenna and reactor, their absorption features, catalytic mechanism, and catalytic efficiency of AR systems could be feasibly tuned.^[^ ^58^, ^59^, ^60^, ^61^, ^62^, ^63^, ^64^ ^]^ In terms of CO_2_ reduction, plasmonic AR systems have been found to improve the catalytic performance for different reaction pathways, such as the reverse water gas shift reaction (RWGS),^[^ ^65^, ^66^, ^67^ ^]^ Sabatier reaction,^[^ ^68^, ^69^, ^70^, ^71^ ^]^ and dry reforming of methane (DRM).^[^ ^72^, ^73^, ^74^, ^75^ ^]^

In this review, we systematically overview recent advancements in heterogeneous CO_2_ reduction by plasmonic bimetallic AR systems. The composition and electronic structures of AR complexes are first illuminated. Subsequently, four plasmon‐mediated catalytic pathways are elaborated, and existing methods are summarized for the deconvolution of distinct mechanisms in synergistic catalysis of CO_2_ reduction by plasmonic AR systems. Finally, challenges and potential future directions for the development of plasmonic AR systems‐based photocatalysis are highlighted. This review provides valuable insights on the structure–function relationship of plasmonic AR systems and in‐depth analyses of their catalytic mechanisms. Under a broader context, it is likely to shed light on the design principles of efficient photocatalysts that further improves solar energy harvesting efficiency toward mitigation of the concurrent environmental crises.

## 2 Composition and Electronic Structures of AR Complex

### 2.1 Composition of AR Complex

**Figure 2:**
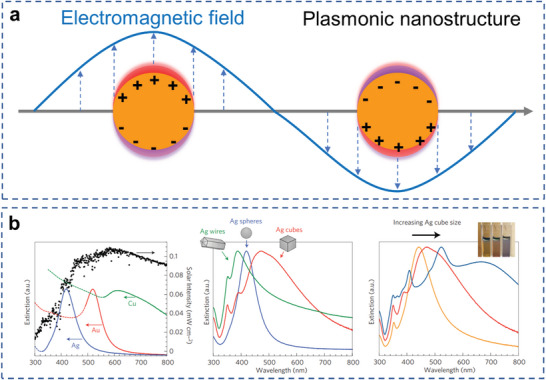
Introduction on LSPR and plasmonic materials. a) Schematic illustration of an excited plasmonic nanoparticle. b) LSPR response of various plasmonic materials with size and geometry. Reproduced with permission.^[^ ^53^ ^]^ Copyright 2011, Springer Nature.

In pioneering works, bimetallic AR systems are a common and intuitive choice of antenna and reactor component, as they allow for synergistic combination of the advantages of the plasmonic and catalytic metals;^[^ ^76^, ^77^, ^78^, ^79^ ^]^ they are also the primary focus of discussions of this review. The optical and photophysical properties of plasmonic AR systems are, on a large scale, derived from the LSPR of the nanoantenna.^[^ ^60^, ^80^, ^81^ ^]^ LSPR describes the collective oscillation of conduction electrons upon resonant illumination at the surface of plasmonic metals, such as Au, Ag, Cu, and recently, Al nanoparticles (NPs) (**Figure** **2**a).^[^ ^50^, ^82^, ^83^ ^]^ The polarizability (*α*) of plasmonic nanoparticles is a crucial character that defines the nanoparticles' response to incoming light.^[^ ^84^, ^85^, ^86^ ^]^ In cases where the particle's size is significantly smaller than the wavelength of light, based on the classical Drude model, the dipolar polarizability *α* can be determined using the Clausius–Mossotti relation as

(1) $$\alpha =\left(1+\kappa \right)V\frac{\varepsilon -\varepsilon_{m}}{\varepsilon +\kappa \varepsilon_{m}}$$

where the dielectric constant of surrounding medium and dielectric function of the metal are denoted by *ε*_m_ and *ε*, respectively, and the volume of the nanoparticle is denoted by *V*. The shape factor *κ* is dependent on the surface geometry. For spherical NPs with radius *R*, one finds *κ*  =  2 and $V=\frac{4}{3}\pi R^{3}$. Therefore, the expression of the NP polarizability in Equation (1) can be adapted for spherical NPs as^[^ ^50^, ^82^, ^87^ ^]^

(2) $$\alpha \;=\;4\pi R^{3}\frac{\varepsilon -\varepsilon_{m}}{\varepsilon +2\varepsilon_{m}}$$

LSPR occurs when *α* is at its maximum for the real part of *ε*  =   − 2*ε*_m_, also known as the Fröhlich resonance condition.^[^ ^88^ ^]^ For a gold sphere with 30 nm size in water, this occurs for *λ* ≈ 530 nm. The classic Drude model is employed to calculate the metallic dielectric function as

(3) $$\varepsilon \;=\;1-\frac{\omega_{p}^{2}}{\omega^{2}+i\gamma \omega }$$

where *ω*_p_ is the bulk plasma frequency of the collective oscillation of free electrons, and *ω* is the angular frequency of the incident light. The system's optical response is collisionally damped with a damping parameter *γ*. The bulk plasma frequency *ω*_p_, which indicates the oscillation frequency of unbound electrons in the bulk, can be expressed as a function of numerous parameters, such as the density of free electrons (*n*), the effective mass of the electrons (*m*_e_), and the permittivity of free space (*ε*_0_), using the equation

(4) $$\omega_{p}=\sqrt{\frac{ne^{2}}{\varepsilon_{0}m_{e}}}$$

LSPR has also been observed in selected transition metals and semiconductor nanocrystals that satisfy the Fröhlich resonance condition.^[^ ^89^, ^90^, ^91^ ^]^ The resonant wavelength and LSPR intensity depend on the size, geometry, and composition of the nanoparticle (**Figure** 2b).^[^ ^49^, ^53^, ^92^ ^]^ By manipulating the nanoparticle, it is possible to design nanostructures that absorb in the entire solar spectrum and beyond.

The excitation of surface plasmon could lead to the localization of electromagnetic (EM) field in regions near the surfaces of plasmonic nanostructures. After excitation, the plasmonic nanoantenna can sustain numerous radiative or nonradiative relaxation pathways, and convert the photon energy into excited carriers and heat in time and space during the relaxation.^[^ ^62^, ^93^, ^94^, ^95^ ^]^ All of them can lead to surface chemical reactivity over plasmonic AR systems, although crucial factors need to be addressed to efficiently harvest the reactivity, such as the rapid decay of EM field intensity, recombination of charge carriers during transfer, and the occurrence of additional decay pathways, which will be elaborated in the following sections.

**Figure 3:**
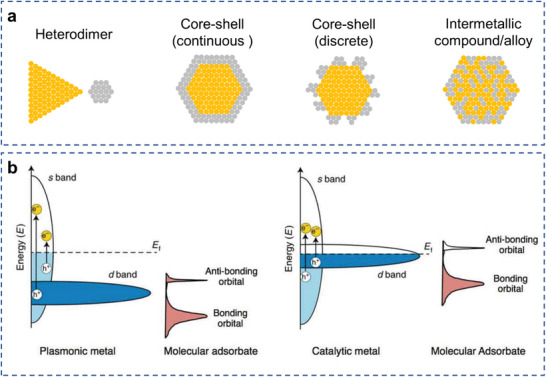
Configurations and electronic structure of plasmonic AR complex. a) Schematic models of distinct structure of plasmonic AR complex: heterodimer, core–shell, and alloyed nanoparticles, with the gold color representing the plasmonic metal and the grey color representing the catalytic metal. b) Electronic band structure of typical plasmonic metals and catalytic metals. Reproduced with permission.^[^ ^80^ ^]^ Copyright 2018, Springer Nature.

Plasmonic AR systems typically occur in three distinct geometries: heterodimer, core–shell, and alloyed nanoparticle systems (**Figure** **3**a).^[^ ^76^, ^96^ ^]^ In 2016, the term of antenna‐reactor system was first used by Prof. Naomi J. Halas and coworkers form Rice University, who reported that the direct coupling of plasmonic Al antennas with catalytic Pd nanoparticles transformed the entire heterodimer complex into an efficient light‐controlled reactive catalyst.^[^ ^56^, ^57^ ^]^ Prof. Zhaoke Zheng and coworkers have also developed a series of core–shell AR structures based on Au nanorod.^[^ ^97^, ^98^, ^99^, ^100^ ^]^ Indeed, in recent years, bimetallic AR catalysts have demonstrated specific selectivity, optical absorption, and enhanced reaction rates for a greater variety of reactions compared to their monometallic counterparts.

### 2.2 Electronic Structure of AR Complex

Plasmonic and catalytic metals derive their properties from inherently different electronic band structures (Figure 3b).^[^ ^80^ ^]^ Plasmonic metals, whose Fermi levels are very much determined by the partially filled s orbitals, own a d‐band far from the Fermi level, lead to intraband electronic transition (s/p to s/p) at resonant frequencies until the onset of interband transitions (d to s/p).^[^ ^82^, ^101^, ^102^ ^]^ In comparison, catalytic metals have d‐band closer to the Fermi level, which leads to interband transitions at optical frequencies.^[^ ^76^ ^]^ Thus, for catalytic metals, their LSPR is dampened by the interband transitions in the whole solar spectrum, which induces a broad spectral feature that is lower in intensity. As a result, enhancement of optical response through introduction of plasmon resonance is typically at the expense of decreasing the catalytic capability.

In order to implement a desirable photocatalyst, both the light absorption capability and the adsorption affinity of gaseous reactants and reaction intermediates are crucial factors to be taken into consideration.^[^ ^12^, ^103^, ^104^ ^]^ However, for a plasmonic system, these two factors could be at a tradeoff with each other. A strong adsorption capacity usually requires a d‐band close to the Fermi level, which is commonly not the case for plasmonic systems. According to the d‐band model of chemical bonding, as the adsorbate approaches a metal surface, its electronic states interact with the sp‐ and the d‐bands of a transition metal at the metal surface.^[^ ^105^ ^]^ The coupling and hybridization of surface sp‐band with the frontier molecular orbitals, such as the highest occupied molecular orbital (HOMO) and lowest unoccupied molecular orbital (LUMO) levels, broadens and shifts the orbital energy.^[^ ^105^, ^106^ ^]^ However, as most transition metals show similar s and p band filling and widths, similar bonding characteristics for the sp‐band coupling can be observed. As a result, the interaction with d‐band is the major cause of the differences in catalytic performance. The surface d‐band that is much narrower than the sp‐states hybridizes with the molecular orbitals, and forms bonding and antibonding states. For plasmonic metals, their d‐bands are typically deep in energy, rendering the antibonding mode after hybridization below the Fermi level, such that the molecule is unable to adsorb to the surface.^[^ ^107^ ^]^ However, importantly, for a catalytic metal, interaction with adsorbates produces antibonding states above the Fermi level, which makes it possible to sustain chemisorption of the adsorbates and thus renders them an effective catalyst.^[^ ^108^, ^109^, ^110^, ^111^ ^]^ At a metal surface, the strength of the interaction between adsorbates and metals depends on the filling of the antibonding states, which, in turn, is determined by their position. Electronic structure leads to the discrepancy in the photoresponse and catalytic effects of plasmonic and transition metals.

Based on the discussions above, the photophysical properties of plasmonic metals and catalytic metals could be complementary. Plasmonic AR complexes created by pairing nanoparticles carrying different intrinsic properties into one entity could utilize the favorable features of both components.

## 3 Photocatalytic CO_2_ Reduction over Plasmonic AR Systems

As discussed above, AR complexes combine the effects from plasmonic and catalytic metals, and may serve as photocatalysts to enhance the efficiency of CO_2_ photocatalysis through multiple mechanisms. In this section, we highlight some examples of AR complex‐based materials for photocatalytic CO_2_ reduction, categorized based on the catalytic mechanisms. The catalyst composition, reaction conditions, and catalytic performance of some reported systems are summarized in **Table** **1**. Major reaction pathways considered in this review are the CO_2_ hydrogenation reactions and CO_2_ reduction with CH_4_ (dry reforming of methane, DRM), which are both industrially valuable but strongly endothermic reactions. It is likely that these reactions can be stimulated effectively through photocatalysis technology based on plasmonic AR systems.

**Table 1:** Summary of AR complexes in CO_2_ photocatalysis

Catalyst	Reaction condition	Light source	Performance	Refs.
Black gold–Ni	CO_2_ hydrogenation	300 W Xe lamp (400–1000 nm)	2464 mmol g^−1^ h^−1^ (CO)	[65]
Au–Ag_8_Cu_1_	CO_2_ hydrogenation	300 W Xe lamp	1867 mmol g^−1^ h^−1^	[112]
Au@Pd	CO_2_ hydrogenation	300 W Xe lamp	3737 mol g^−1^ h^−1^ (CO)	[113]
Cu@Co	CO_2_ hydrogenation	300 W Xe lamp	920.28 µmol g^−1^ h^−1^	[114]
Ag–Ni	CO_2_ hydrogenation	405 nm laser	26.7% (CO_2_)	[68]
Au–Ni	CO_2_ hydrogenation	520 nm laser	34% (CO_2_)	[69]
Au–Cu	CO_2_ hydrogenation	Xe lamp	86.9 µmol g^−1^ h^−1^	[115]
Au&Pt@ZIF	CO_2_ hydrogenation	Xe lamp	1522 h^−1^	[116]
Al@Cu_2_O	CO_2_ hydrogenation	Laser (400–850 nm)	360 µmolcm^−2^ h^−1^ (CO)	[67]
Cu–Ru	DRM	Laser	34 mol H_2_ (mol Ru) ^−1^ s^−1^	[75]
Pt–Au/SiO_2_	DRM	Xe lamp (300–800 nm)	68.6 µmol g^−1^ min^−1^ (CO_2_)	[72]
Pd_90_Au_10_/Al_2_O_3_	DRM	Xe lamp	1210.6 µmol g^−1^ min^−1^ (CO_2_)	[74]
Rh–Au/SBA‐15	DRM	Xe lamp	3600 mmol g^−1^ s^−1^ (CO_2_)	[73]

For plasmonic AR systems, three major reaction pathways could prove effective in CO_2_ photocatalysis based on distinct plasmon‐induced photophysical processes, which are respectively plasmon‐induced resonant energy transfer mechanism, hot charge carrier‐mediated catalytic mechanism, and photothermal catalytic mechanism. Since the various nonradiative relaxation pathways can happen simultaneously following the photoexcitation of the AR systems, multiple reaction pathways could coexist synergistically. In this section, specific works in CO_2_ photocatalysis over plasmonic AR systems are categorized based on their distinct catalytic mechanisms.

### 3.1 Plasmon‐Induced Resonant Energy Transfer Mechanism

When illuminated, the plasmonic nanoparticles can sustain a collective resonant oscillation of free charge about the metal surfaces, leading to the formation of a localized EM field.^[^ ^117^, ^118^, ^119^, ^120^ ^]^ The intensity of EM field is highest on the surface of plasmonic structure, but decays exponentially with increasing distance from the surface. With the significantly increased optical cross‐sections,^[^ ^121^, ^122^ ^]^ plasmonic nanoparticles are likely to drastically enhance the absorption processes associated with the inter‐ or intraband transition modes and thus the photoexcitation of quantum emitters, photosensitizers, or photocatalyst molecules or particles localized within the EM field.^[^ ^123^ ^]^ In addition, the presence of these components within the vicinity of plasmonic metal nanoparticles is likely to increase the local density of electromagnetic states (LDOS), leading to further broadening of the absorption features. In the cases where the absorption processes of the photocatalyst components are in resonant frequencies with the LSPR frequency of the plasmonic metal, even more evident enhancement in the absorption and thus, the photoexcitation processes could exist through constructive interference of the two resonant processes.^[^ ^39^, ^124^, ^125^ ^]^ These effects could lead to a 10^3^‐ to 10^5^‐fold enhancement of the photoexcitation rate, while the enhancement factors (EFs) could be further increased by a few orders of magnitude through enhancement by narrow junctions formed between multiple plasmonic particles, referred to as plasmonic hot spots, where extremely high‐intensity fields can be observed.^[^ ^126^ ^]^ This enhanced EM field results in an increased probability of excitation of the reactant or substrate as well as increased excited state population, which could directly facilitate the catalysis of photochemical reactions.

**Figure 4:**
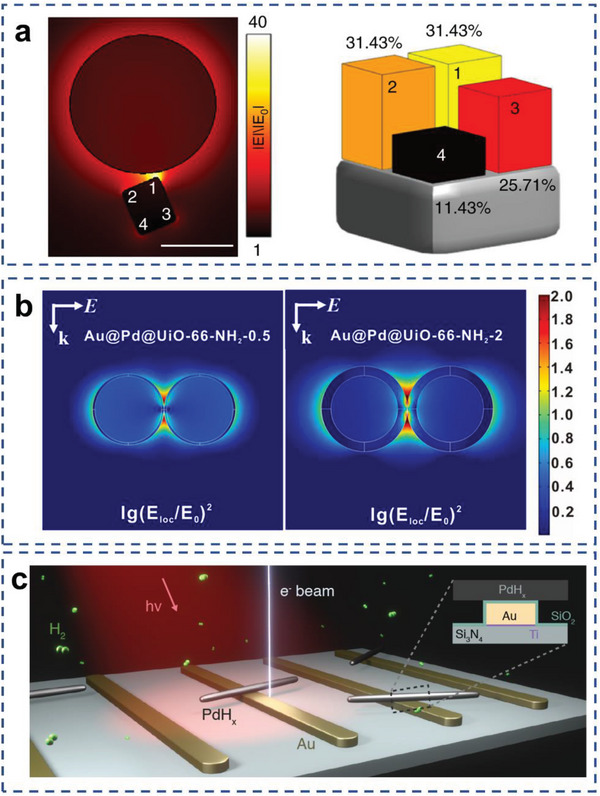
Applications of AR complex over field‐enhanced mechanism. a) Calculated field enhancement for Au‐Pd AR system. Reproduced with permission.^[^ ^95^ ^]^ Copyright 2018, Springer Nature. b) Enhanced electric field distributions of Au@Pd nanoparticles. Reproduced with permission.^[^ ^66^ ^]^ Copyright 2021, Elsevier Ltd. c) Schematic illustration of optically coupled E‐TEM measurement over Au–Pd crossed‐bar nanostructure. Reproduced with permission.^[^ ^133^ ^]^ Copyright 2021, American Association for the Advancement of Science.

From classical electrodynamics, plasmonic enhancement is inversely related to interparticle distance, as the resonant electromagnetic field decays exponentially as the distance increase. The gap between antenna and reactor should be as small as possible to increase photocatalytic reactions (**Figure** **4**a).^[^ ^95^ ^]^ However, when the two metals are brought into close proximity, quantum effects such as localization of surface charges and tunneling, as well as quenching of surface excited states through additional nonradiative relaxation pathways will decrease the plasmonic enhancement.^[^ ^127^, ^128^, ^129^, ^130^, ^131^, ^132^ ^]^ Therefore, an optimized distance range exists to balance the tradeoff between resonant field‐enhancement and quenching or additional loss pathways. When this gap shrinks to atomic scales to form core@shell AR nanoparticles, in which the plasmonic metal core is surrounded by a thin (continuous or discrete) catalytic shell, the situation becomes completely different (**Figure** 3a). Meanwhile, disparate shell metals with high work functions can be introduced as efficient electron acceptors for enhancing charge separation.

Previous researches mainly focused on the plasmonic particle/semiconductor^[^ ^53^, ^134^ ^]^ and plasmonic/metal complex systems^[^ ^127^, ^135^, ^136^, ^137^ ^]^ that can lead to chemical reactions in specific regions or enhance light absorption and inhibit the electron–hole recombination. Recently, the field‐enhancement mechanism has been exploited by AR complex in photocatalytic CO_2_ reduction based on H_2_ as reductant. Zhang et al. designedly prepared an AR plasmonic catalyst with novel MOFs encapsulated Au@Pd nanoparticles for light driven CO_2_ hydrogenation under mild conditions.^[^ ^66^ ^]^ Under illumination by a 300 W Xe lamp, CO_2_ was converted to CO with high selectivity. The Au core acts as an antenna to generate strong localized EM field, as well as enhances the near field of the Pd shell (Figure 4b). The ultrathin Pd shell significantly improved the catalytic activity. Ultrafast transient absorption (TA) spectroscopy data and the theoretical calculations revealed that the coupling of EM field and thermal electron reduce the reaction energy barrier from CO_2_* to COOH*.

Besides, the resonant energy transfer mechanism could also promote photochemical catalysis by affecting the chemisorption of reactants or reaction intermediates, and by inducing chemical bond activation through dissipation of field energy into bond vibrational energy.^[^ ^138^, ^139^, ^140^ ^]^ Although these mechanisms have not been utilized for CO_2_ photocatalysis to the best of our knowledge, yet it presents great promises for lowering the activation energy barrier and facilitating such reactions. For instance, Li et al. evidenced that Ag nanoparticle will enhance Pt light absorption in the AR configuration.^[^ ^61^ ^]^ Meanwhile, the enhanced near field at the Pt—CO interface due to localization near Ag increased the rate of direct photoexcitation of the Pt—CO bond and thus the rate of CO oxidation. Xiong and coworkers reported surfaced plasmon mediated nitrogen fixation using Au—Ru AR nanostructure as catalyst.^[^ ^141^ ^]^ In the enhanced EM field, N_2_ molecule can be well chemisorbed at Ru sites to form Ru–N_2_ complex near plasmonic Au. This hybrid state can allow direct excitation of the carrier in the complex by the surface plasmon to induce N_2_ dissociation and form chemisorbed ≡N. Recently, Prof. Jennifer A. Dionne and coworkers reported the dehydrogenation on crossed‐bar Au–Pd antenna‐reactors, where the plasmon‐enhanced local EM‐field enhancement is localized away from the Pd nanorod tips.^[^ ^133^ ^]^ By tracking the dehydrogenation process as the function of light intensity, illumination wavelength, and hydrogen pressure, they found that plasmons could shift dehydrogenation away from the sharp Pd nanorod tips to the flat middle faces (Figure 4c). This work has further improved the understanding of the field‐enhancement mechanism.

### 3.2 Hot Carrier‐Mediated Reaction Mechanism

**Figure 5:**
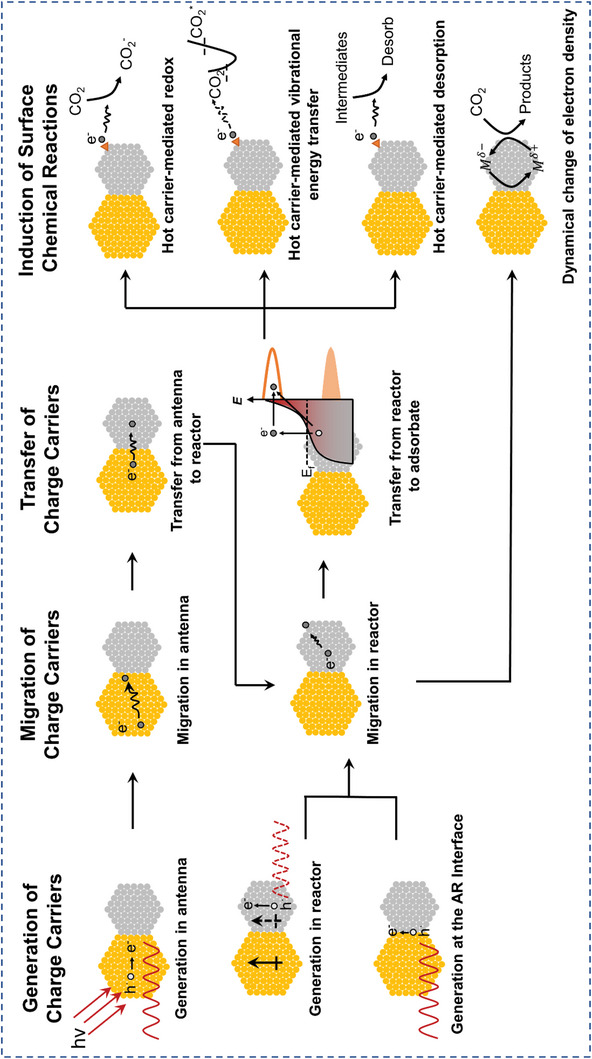
General scheme of hot carrier‐mediated catalytic pathways of plasmonic AR systems.

Plasmonic hot charge carrier‐mediated catalysis is one major catalytic mechanism for plasmonic AR systems. Charge carrier‐induced reactivity can typically occur in several consecutive steps, namely the generation, migration, transfer of charge carriers, and initiation of surface chemical reactivity (**Figure** **5**). After photoexcitation of the nanoantenna, the nonradiative damping can induce excitation of charge carriers through electronic intraband and/or interband transitions,^[^ ^142^, ^143^ ^]^ followed by thermalization of charge carrier energy through electron–electron scattering into higher‐temperature Fermi–Dirac distributions.^[^ ^144^, ^145^ ^]^ This process can prepare hot electrons at energy levels above the Fermi energy (*E*_f_) of the metal and/or hot holes below *E*_f_.^[^ ^146^ ^]^ This is followed by the migration of hot carriers to the surface of the metal NPs, overcoming the work function;^[^ ^147^ ^]^ as well as possible transfer to the catalyst component or surrounding molecular adsorbates through indirect or direct transfer pathways. Subsequently, the hot carriers will be capable of driving electronic and chemical processes at the surface of the NPs through photochemical redox pathways.^[^ ^49^, ^107^ ^]^

#### 3.2.1 Generation and Migration of Hot Carrier

For a number of AR catalysts, hot‐carrier‐driven process is the main reaction mechanism. For different configurations of AR catalysts, hot carriers are generated at different locations and thus have different migration behaviors. Here we briefly summarize the generation and transfer of hot carrier based on AR complex, and how they facilitate photocatalytic reactivity.

##### Charge Carrier Generation in Antenna

**Figure 6:**
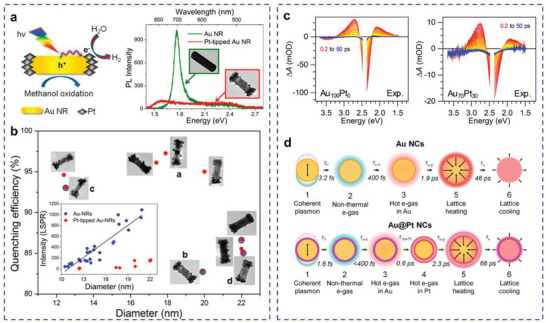
Charge carrier generation in plasmonic antenna. a) UV–vis–NIR extinction spectra and PL spectra of Pt‐tipped Au nanorods. b) PL quenching efficiency of individual Pt‐tipped Au nanorods. Reproduced with permission.^[^ ^148^ ^]^ Copyright 2014, American Chemical Society. c) Experimental transient absorption spectra from 0.2 to 50 ps for Au_100_Pt_0_ and Au_70_Pt_30_ catalysts. d) Schematic sequence and time constants for the Au and Au@Pt. Reproduced with permission.^[^ ^149^ ^]^ Copyright 2020, American Chemical Society.

Through photoexcitation of the LSPR, hot charge carriers can be excited in the plasmonic nanoantenna components.^[^ ^118^ ^]^ Zheng et al. analyzed the quenching efficiency of individual Pt‐tipped Au nanorods with detailed morphological information to understand the energy relaxation path of plasmon‐generated hot electrons.^[^ ^148^ ^]^ The strong quenching of photoluminescence (PL) and darkfield scattering spectra at the LSPR region confirmed the hot carrier production from Au nanorods (**Figure** **6**a,b). Apkarian and coworkers investigated the structure and ultrafast photodynamics of Au@Pt AR photocatalyst with ultrathin Pt shell.^[^ ^149^ ^]^ The ultrafast transient absorption data indicated that after photoexcitation, the energy transfers from the plasmonic core to the Pt shell in the form of electron flow and back to the lattice of the core in the form of heat. The strong coupling of Pt electrons led to accelerated dephasing of the Au plasmon on the femtosecond time scale. Then the hot carriers generated in the Au core were entirely dumped into the Pt shell within a few sub‐picoseconds, which drove the reaction before losing energy to the lattice (**Figure** 6c,d). Strong broadband optical absorption, enhanced EM fields and effective transfer of hot carriers to the catalyst surface make Au@Pt AR nanocrystals an efficient photocatalyst.

##### Charge Carrier Generation in Reactor

**Figure 7:**
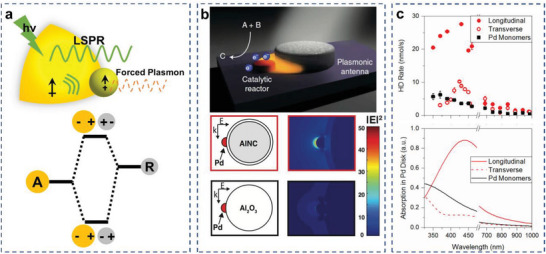
Forced plasmon in the AR photocatalysts. a) Schematic model of forced plasmon and hybridization diagram of plasmonic AR system. b) Generalized scheme of an AR system and near‐field enhancement in Al‐Pd catalysts. Reproduced with permission.^[^ ^56^ ^]^ Copyright 2016, AAAS. c) Wavelength‐dependent HD production rate and absorption of Pd disk in Al‐Pd AR photocatalysts. Reproduced with permission.^[^ ^57^ ^]^ Copyright 2016, American Chemical Society.

When two dissimilar metals are in proximity, their respective primitive plasmonic excitations result in the interaction of local fields, thereby forming hybridized bonds and antibonded plasmonic excitations.^[^ ^150^ ^]^ The adiabatic molecular orbital theory posits that the bonding mode is a blend of the primordial plasmon of the two particles, which is dominated by the lower energy primordial plasmon, such as the longitudinal mode of the LSPR. Conversely, the higher energy primordial equipartition excitations, such as the transverse equipartition exciton resonance mode, contribute more significantly to the antibonding mode. In the diabatic representation, the optical resonance of this kind of heterodimer is dominated by the plasmonic component.^[^ ^151^ ^]^ The optically poor nanoparticle (catalytic metal) is driven by the wavelength and polarization‐dependent near field of the plasmonic nanoparticle, creating an induced dipolar oscillation and therefore a plasmonic response in it. This induced plasmon oscillation mode has been referred to as “forced plasmon” (**Figure** **7**a).

The forced plasmon mechanism drastically enhances the optical cross‐sections of the catalytic nanoparticle. When both nanoparticles have strong plasmonic responses, even when they are in different wavelength ranges, forced plasmon can also be excited. The near field of the on‐resonance nanoparticle can force a polarization in the off‐resonance nanoparticle, creating a plasmon resonance at similar frequencies subject to nonradiative energy relaxation. Based on the phase analysis for the forced harmonic oscillator, the two nanoparticles will oscillate in phase for the lower energy mode, and out of phase for the higher energy mode. When this “forced plasmon” decays, it can generate hot carriers in catalytic metals, making it a photocatalyst in addition to maintaining its innate catalytic properties.^[^ ^56^, ^57^, ^59^, ^61^, ^152^, ^153^, ^154^ ^]^

Halas and coworkers demonstrated forced plasmons for photocatalysis with Al–Pd AR photocatalysts. The Al core was surrounded by an intrinsic 2–4 nm self‐limiting oxide, separating it from Pd islands grown directly onto the outside of the Al_2_O_3_ shell, which also limited the possibility of carrier migration from Al to Pd. When laser illuminated, the plasmonic Al induced an optical polarization through its optical near field, driving a plasmon in the Pd islands (**Figure** 7b).^[^ ^56^ ^]^ The wavelength dependence of HD production closely followed the calculated absorption cross‐section and the reaction rate was super‐linearly related to the light intensity, which served as evidence for a hot‐carrier‐driven reaction. To better understand this AR modulation mechanism, finite‐difference time‐domain (FDTD) simulations were performed to calculate the optical absorption in the Pd disk for the AR photocatalysts and the isolated Pd monomers. For longitudinal polarization, the absorption of the Pd disk in the AR photocatalysts was enhanced by the near field of the Al disk compared with the Pd monomer. This was in agreement with the enhancement of the HD generation rate observed experimentally for longitudinal polarization (**Figure** 7c).^[^ ^57^ ^]^

##### Charge Carrier Production at the Plasmonic/Nonplasmonic Interface

**Figure 8:**
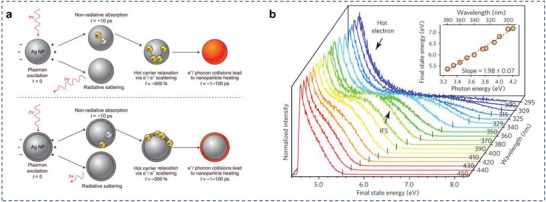
Charge carrier production at the plasmonic/nonplasmonic interface. a) Illustration of sequential plasmon excitation and decay processes in: an illuminated monometallic Ag nanoparticle and Ag nanoparticle with another material attached to the nanoparticle surface. Reproduced with permission.^[^ ^52^ ^]^ Copyright 2021, Springer Nature Limited. b) A series of normalized 2PP spectra of the Ag/TiO_2_ surface with excitation wavelengths between 450 and 295 nm. Reproduced with permission.^[^ ^156^ ^]^ Copyright 2017, Springer Nature.

Due to the formation of the chemical bonds between nonplasmonic shell and the plasmonic metal, local interfacial electronic states arise that are shared between these two materials. In most cases, the interfacial state allows direct momentum conserved excitations. Thus, Linic and coworkers postulated that the initial energetic e–h pair could be generated at the plasmonic/nonplasmonic interface within the AR nanostructure. This reasoning has critical consequences on the flow and dissipation of energy within the AR material (**Figure** **8**a).^[^ ^52^ ^]^

This mechanism have recently been tested in a number of measurements on a single nanoparticle level. Lian and coworkers showed the first indications in their study that plasmons were able to decay directly into interfacial electron–hole pairs.^[^ ^155^ ^]^ With the aid of the model of cadmium selenide nanorods modified gold, they found that the gold plasmon was strongly damped by cadmium selenide through interfacial electron transfer. Tan et al. used ultrafast two‐photon photoemission (2PP) spectroscopy to measure the LSPR decay with high spatial and temporal resolution in Ag/TiO_2_ AR system.^[^ ^156^ ^]^ Hot carrier production through interface‐mediated processes is typically much faster (in several fs) than those induced by dephasing of the excited plasmons and thermalization processes. These hot electrons almost exclusively originated from the interfacial electronic states between Ag and TiO_2_ (**Figure** 8b). The results supported the notion that the interfacial Ag/TiO_2_ states opened up fast energy dissipation channels and that the location of the initial energetic e–h formation proceeded through these interfacial states. Similar results were observed by Foerster et al. Plasmon decay rates were measured for each interface by evaluating the resonance line width in the scattering spectra of single gold nanorods.^[^ ^157^ ^]^ The conventional hot electron decay of the same interfaces was investigated by 2PP on continuous gold films, which provided the density and lifetime of unoccupied states. The measurements showed that the LSPR decay rate was governed by the availability of energetically accessible electronic states at the interface of Au and metal oxides, which indicated that the decay proceeded through these interfacial states.

However, there is still a lack of studies on the initial formation of energetic e–h pairs in the plasmonic/nonplasmonic interfaces in bimetallic AR systems. Besides, given the much shorter lifetimes for the interface‐mediated hot carriers, their compatibility and applicability for charge transfer‐induced reactions remains to be investigated. Therefore, additional research is necessary to rigorously assess the geometric locations on the initial formation of energetic charge carriers.

#### 3.2.2 Transfer of Hot Carrier

**Figure 9:**
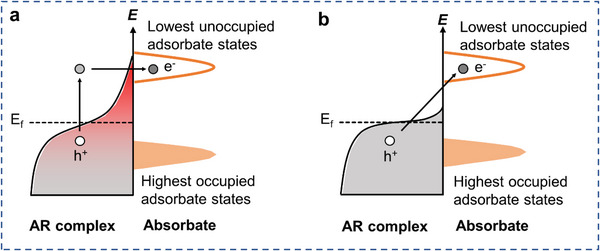
Schematic illustrations of the hot carrier transfer pathway. a) The indirect hot carrier transfer mechanism. b) The direct hot carrier transfer mechanism.

After the generation of charge carriers and migration to interfaces, the hot carriers can then transfer via indirect or direct electron transfer (**Figure** **9**). This categorization of hot carrier transfer is general and applies to various scenarios, such as from antenna to reactor and from reactor to the acceptor level of adsorbate prior to induction of chemical reactions.^[^ ^155^, ^158^, ^159^ ^]^

##### Indirect Hot Electron Transfer into the Adsorbate

After plasmon excitation, hot electrons are generated within the catalytic nanoparticle or the interface, thermalized through electron–electron scattering, and transferred to the lowest unoccupied molecular orbital (LUMO) of the adsorbed species on the catalyst surface (Figure 9a). Generation of kinetically excited charge carriers for indirect transfer processes is in general favored by smaller antenna nanoparticles with diameters (*d*) < 20 nm and strong E‐field intensity.^[^ ^52^, ^160^ ^]^ The efficiency also depends on the imaginary part of the dielectric function of the metal and increases in wavelength ranges where interband transitions are accessible.^[^ ^161^ ^]^ Transient absorption results in previous studies have demonstrated the thermalization of electrons in Au and subsequent cooling process, which evidences that the hot electrons transfer through an indirect transfer pathway (Figure 7c). Since the transfer of electrons to the adsorbate occurs after the hot carrier generation, it is limited by the energy loss due to electron–electron scattering. The efficiency of indirect hot electron transfer is positively correlated with the incident photon energy. With a higher incident energy, the electrons could be thermalized into higher‐temperature Fermi–Dirac energy distribution, which contains a larger proportion of high‐energy electrons. Thus, the probability of electron injection into the LUMO of adsorbates will be greatly increased.^[^ ^162^, ^163^, ^164^, ^165^ ^]^

##### Direct Hot Electron Transfer into the Adsorbate

The hot electrons can also be exploited for catalytic applications by direct injection from metal nanoparticles to adsorbates.^[^ ^48^, ^166^ ^]^ This plasmon dephasing pathway is caused by the coupling between unoccupied adsorbate states and the excited surface plasmons.^[^ ^167^, ^168^ ^]^ Therefore, energy matching between the adsorbate acceptor energy levels and the Fermi level of the plasmonic metal is typically required. Hot electrons are directly transferred into the hybridized states between the catalyst nanoparticles and adsorbed species during the direct transfer process (Figure 9b). The efficiency of direct transfer is proportional to the probability that a surface‐scattered electron is transferred to the adsorbate as well as the Fermi velocity. It is inversely proportional to the effective electron path length, which depends on the size and morphology of the particle.^[^ ^169^ ^]^ Unlike the indirect mechanism, which occurs after separation of charge carriers and thermalization, the direct electron transfer occurs simultaneously during the plasmon excitation dephasing process. Thus, the direct electron transfer mechanism is expected to exhibit higher efficiency and smaller energy loss due to the bypassing of electron–electron scattering. However, the occurrence of direct electron transfer typically has a low probability because it requires strong adsorbate/metal interactions to achieve surface orbital hybridization, which is uncommon in plasmonic photocatalysts.^[^ ^155^, ^158^ ^]^

#### 3.2.3 Induction of Surface Chemical Reactions

Following the localization of hot carriers at the surfaces of the reactor components in the plasmonic AR systems, it is likely that they could initiate chemical conversions.^[^ ^144^, ^167^, ^170^ ^]^ Through injection of charge carriers into the adsorbate, direct redox catalysis could take place. In addition, injection of hot carriers could also lead to desorption of reaction intermediates or products, significantly affecting the reaction pathways and product selectivity. Furthermore, transient charge transfer toward the adsorbate has also been proposed where the carriers decay through conferring their energy into the vibrational energy of the adsorbate molecules, promoting them to higher vibrational states, activating subsequent chemical conversions. It is worth noting that the electron density of the metal could also be affected by the hot carriers, which can either directly affect the reduction performance, or in turn, lead to additional consequences including the above mechanisms.

##### Inducing Direct Redox Photocatalysis through Injection of Charge Carriers

**Figure 10:**
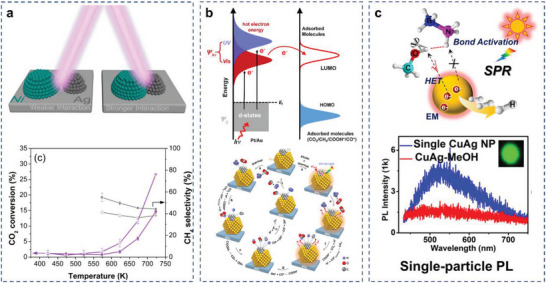
Direct redox photocatalysis mechanism. a) Reaction scheme and CO_2_ conversion and methane selectivity for Ag‐Ni/SiO_2_. Reproduced with permission.^[^ ^68^ ^]^ Copyright 2020, American Chemical Society. b) DRIFTS spectra of Au‐Pt/SiO_2_ and Pt/SiO_2_ measured under dark condition and with light. Reproduced with permission.^[^ ^72^ ^]^ Copyright 2017, American Chemical Society. c) Proposed mechanism for plasmon‐enhanced AB methanolysis in the presence of the Cu–Ag AR photocatalysts. Reproduced with permission.^[^ ^175^ ^]^ Copyright 2021, American Chemical Society.

In terms of CO_2_ photocatalysis, direct injection of hot carriers has produced numerous examples of CO_2_ hydrogenation or DRM catalysis. For instance, Ag–Ni AR photocatalysts were synthesized by Rose Amal and coworkers to modulate the interaction between the plasmonic and reactive metals and to examine the impact of visible light irradiation on the methanation of CO_2_. Ni and Ag were sequentially impregnated on SiO_2_ support, which provided a stronger bimetallic interaction as well as charge transfer, evidenced by X–ray photoelectron spectroscopy（XPS） studies. The increase in CO_2_ conversion under laser irradiation was attributed to the contribution of the Ag photoresponse. (**Figure** **10**a).^[^ ^68^ ^]^ This work also studied the metal interaction and its subsequent impact on the catalytic performance and propensity toward light enhancement by systematically modulating the metal deposition protocol.

Ye and coworkers have made many attempts in photocatalytic DRM reaction through the hot carrier‐induced pathway.^[^ ^72^, ^73^ ^]^ For example, they reported for the first time that visible light‐induced Au LSPRs have the ability to activate CO_2_ and CH_4_. Under irradiation, the highly energetic electrons generated by Au can effectively enhance the catalytic performance of Rh/SBA‐15 in DRM. In addition, they demonstrated the coupling of Au and Pt for efficient CO_2_ reduction in DRM reduction. Under low‐intensity light (300–800 nm, 0.6 W cm^−2^) irradiation, the activation energies for CO_2_ reduction are reduced 30% below thermal activation energies, and the reaction rate is 2.4‐times higher than that of the thermal‐catalytic reaction rate at 673 K (**Figure** 10b).^[^ ^72^ ^]^ FDTD simulations, density functional theory (DFT) calculations and in situ diffuse reflectance infrared Fourier transform spectroscopy (DRIFTS) experiments demonstrated that the strong plasmonic coupling of Au and Pt generated stronger electric fields and excited high concentrations of hot carriers, which activated the adsorbed reactants and intermediate species, and increased the reaction rate. Apart from that, they found that metal nitrides, such as TaN,^[^ ^171^ ^]^ were potentially plasmonic materials, which is likely to greatly expand the material composition of AR catalysts.

Production of hot carriers could modify the reaction pathway, enhance the selectivity and suppress unintended side reactions.^[^ ^172^, ^173^, ^174^ ^]^ Many of these studies are achieved by injection of hot carriers into the antibonding orbitals of adsorbates and therefore, activation of the photocatalysis of reactions that involves chemical bond cleavage. In one of these studies, Ag–Cu AR photocatalysts with controlled compositions exhibit an excellent H_2_ yield under continuous‐wave (CW) light illumination for ammonia borane methanolysis reaction. The single‐particle study combined with DFT simulations showed that the O—H bond in CH_3_OH molecule was activated by hot electrons. Then, CH_3_OH* relaxed by releasing an electron back to the metal, acquiring vibrational energy, and finally, leading to decomposition (**Figure** 10c).^[^ ^175^ ^]^ Meanwhile, Zheng and coworkers have also designed a variety of AR photocatalysts for application in different reactions, such as Au–Pd nanorods for selective hydrogenation of nitrobenzene and Au–Pt nanorods for ammonia borane dehydrogenation.^[^ ^97^, ^98^ ^]^ The hot carriers transfer from photocatalysts to reactant was confirmed as the key factor for the plasmon‐enhanced catalytic activity. The energetic charge carriers can efficiently couple to the adsorbed reactant molecule, providing sufficient energy to activate specific chemical bonds and directly drive catalytic reactions. Tian and coworkers confirmed hot carriers in AR photocatalysts could promote oxygen activation and regulated the reaction path to improve the acrolein selectivity while decreasing the propylene oxide selectivity under illumination for the propylene partial oxidation reaction. This mechanism does not seem to have been reported in the work on AR‐catalyzed CO_2_ reaction and remains to be explored.^[^ ^176^ ^]^

##### Accelerating the Desorption of Certain Surface‐Adsorbed Species

Injection of hot carriers into adsorbate molecules’ unoccupied orbitals could also promote the desorption of surface adsorbates, affecting reaction pathways on a large extent. By DRIFTS, Song et al. demonstrated that intensities of the characteristic peaks of CO species obviously decreased under light illumination, resulting in the lower surface coverage of absorbed CO species.^[^ ^72^ ^]^ The lower species coverage meant that the adsorbed CO species were more easily desorbed to form CO and H_2_ under light irradiation, which could explain the enhancement of catalytic activities and the lower activation energy in DRM reaction.

**Figure 11:**
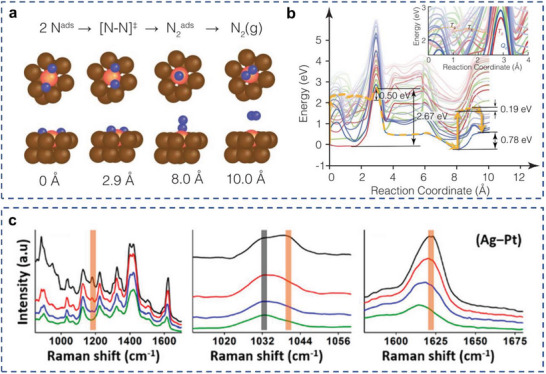
Different mechanisms of surface chemical reactions. a,b) Plane‐wave density‐functional theory for N_2_ dissociative adsorption on Cu–Fe AR catalyst surface. Reproduced with permission.^[^ ^178^ ^]^ Copyright 2022, AAAS. b) Stokes spectra for MB/Ag–Pt samples collected at different time intervals. Reproduced with permission.^[^ ^179^ ^]^ Copyright 2019, American Chemical Society.

This mechanism of promoting desorption is also commonly seen in other reactions catalyzed by AR complexes. Halas and coworkers demonstrated efficient and stable plasmon‐driven hydrodefluorination of CH_3_F on an Al–Pd AR photocatalyst.^[^ ^177^ ^]^ They evidenced that photogenerated carriers are likely to contribute to cleaning the active sites via D_2_ desorption from the Pd surface, which leads to an enhanced reactivity under illumination for acetylene hydrogenation. Plasmon‐induced hot carriers led to rapid desorption of H_2_, and thus limiting the availability of hydrogen on the surface for additional hydrogenation of ethylene. Recently, they further demonstrated that plasmonic AR systems can transform the thermally unreactive transition metal, Fe, into a catalytically active site under illumination. Fe active sites in the Cu‐Fe AR complex achieve efficiencies very similar to Ru for the photocatalytic decomposition of ammonia under ultrafast pulsed illumination.^[^ ^178^ ^]^ Embedded correlated wave function theory was used to calculate potential energy surfaces (PESs) projected along minimum‐energy paths. The associative desorption of N_2_ on Cu‐Fe AR catalyst was experimentally observed to be the rate‐determining step (**Figure** **11**a,b). The hot carriers stimulated the formation of adsorbate‐metal excited states, activated the metal‐adsorbate bond, decreased the apparent and elementary step activation barriers, cleaned occupied active sites, and facilitated product desorption, thereby enhancing both the reactivity and stability of Cu–Fe AR catalyst.

##### Hot Carriers‐Mediated Vibrational Energy Transfer

Recently, a novel charge carrier‐induced energy transfer mechanism, termed transient charge transfer, has been proposed and characterized. Instead of direct extraction and injection of charge carriers into adsorbate unoccupied orbitals, this mechanism involves the transfer of charge carrier energy into the vibrational energy of the adsorbate molecule, and thus facilitating adsorbate activation. Linic and coworkers proposed and evidenced this effect with a Ag–Pt core–shell catalyst toward the catalytic reduction of methylene blue (MB) through in situ Raman spectroscopy, where high anti‐Stokes scattering intensities and a blueshift in Stokes modes were observed (**Figure** 11c).^[^ ^179^ ^]^ They concluded that the transient charge transfer leads to higher vibrational energies of MB, which activates it for chemical reduction reaction. Further characterization at transient timescales for this mechanism could be helpful to deepen its understanding and broaden its application in plasmonic AR systems toward CO_2_ reduction reactions.

##### Dynamical Change of Electron Density

The generation and aggregation of hot carriers in the catalytic component could also affect the electron density of the metal, which can either directly affect the reduction performance or lead to additional consequences including the above (1)–(3). In terms of CO_2_ reduction, the key factor that affects gas‐phase CO_2_ hydrogenation selectivity is the binding energy of the intermediates (e.g., CO), which is closely related to the electronic properties of the active metal. Catalysts with high electron density adsorb CO strongly, which could lead to the further hydrogenation of CO into CH_4_; while catalysts with low electron density favor CO desorption.^[^ ^180^ ^]^ On the other hand, the electron density may also bring about changes in the valence state of the metal. Reinhard and coworkers integrated Au nanorods with a longitudinal LSPR at 745 nm and CuFeS_2_ nanocrystals with a resonance peak at 490 nm for robust photocatalysis of hydrogen evolution reactions (HER) in a wavelength‐dependent manner. The excess electrons on CuFeS_2_ are available for reducing any surface‐available Cu(II) back to Cu(I), which can, in turn, be coupled to proton reduction while being oxidized back to Cu(II).^[^ ^40^ ^]^ However, the applicability of this mechanism in CO_2_ reduction photocatalysis remains to be investigated.

**Figure 12:**
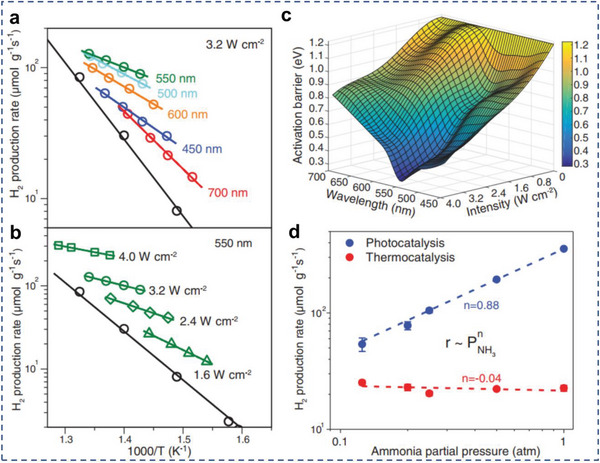
Reducing activation barrier for certain reaction steps. Arrhenius plots of apparent activation barriers for a) different wavelengths under constant intensity and b) various light intensities at 550 nm. c) A 3D representation of *E*_a_ for different wavelengths and intensities. d) Reaction order with respect to P_NH3_ in photocatalysis and thermocatalysis. Reproduced with permission.^[^ ^182^ ^]^ Copyright 2018, AAAS.

One common aspect for all mechanisms described above is that they are likely to reduce the activation energy barrier for the catalyzed reactions. Previous reviews outlining the mechanisms of plasmon‐mediated photocatalysis in reducing activation barrier mechanism are already available. In one of these studies, Kim et al. demonstrated ethanol dehydrogenation under light irradiation using Ag–Ni AR photocatalysts.^[^ ^181^ ^]^ The absorbed light energy was transferred to the catalytic component by the surface plasmon through the hot electrons produced in nonradiative decay pathways. The effective energy barrier was greatly reduced from 41.6 to 22.3 kJ mol^−1^ for the catalyst under light irradiation. Halas and coworkers reported the relationship between hot‐electron excitation and plasmonic heating effect in a recent study of plasmon‐driven NH_3_ decomposition reaction, where the concept of “light dependent activation barrier” was raised for the first time.^[^ ^182^ ^]^ It was found in this work that for a Cu–Ru catalyst, the thermal catalytic activity was much lower than the photocatalytic activity, indicating that energetic electron played a crucial role in the reaction. In order to quantify the contribution of hot electrons, the authors further calculated light‐dependent activation barrier by measuring the Arrhenius plots at different light intensities and wavelengths (**Figure** **12**). The introduction of hot electron was demonstrated to reduce the energy barrier of the rate‐limiting step in thermally driven NH_3_ decomposition using DFT calculations. The hot electrons could induce multiple vibrational transitions of the reactant molecule, and as the vibrational energy stored in the bond increases, the activation energy was reduced. This pioneering work sheds light on the deconvolution of complicated LSPR effect in photocatalysis. However, it is also noteworthy that in many cases, this method alone is often insufficient to completely rule out thermal contributions to the measured rate enhancements. Additional experimental evidence for the role of nonthermal effects in plasmon‐driven photocatalysis is typically necessary.^[^ ^183^ ^]^

### 3.3 Photothermal Process

As discussed above, one most common approach of photothermal heating for AR systems is through electron–phonon scattering. Under resonant conditions, charge carrier separations are induced through nonradiative relaxation of the excited plasmon from 1 to 100 fs. At longer relaxation times, electron–electron scattering occurs over a period from 100 fs to 1 ps. This is followed by electron–phonon scattering and increasing in the temperature of the metal lattice with a timescale of several to hundreds of ps. Subsequently, through phonon–phonon scattering, this heat is transferred to the surroundings in the time scale from 100 ps to 10 ns. With pulsed irradiation at shorter pulses (<0.1 ns) and/or when the plasmonic nanoparticle is small enough (*d* < 100 nm), these steps are considered to happen consecutively.^[^ ^37^, ^43^, ^184^, ^185^ ^]^

Although for plasmonic AR system, additional photothermal heating mechanisms, such as mechanical heating through plasmon‐mediated shockwave generation, chemical heating through excited carrier‐induced exothermic reactions, and heating through direct dephasing of excited plasmons into bond vibrational energy could also play a role;^[^ ^186^, ^187^, ^188^ ^]^ yet considering the specific design and illumination conditions in a vast majority of works that utilize AR systems toward photocatalysis, the hot electron‐induced heating pathway introduced above could have major contribution.^[^ ^189^, ^190^, ^191^, ^192^, ^193^, ^194^ ^]^ It has been suggested in previous literatures that, with CW irradiation, the released heat induced by electron–phonon scattering is correlated with various parameters according to Equation (5).^[^ ^86^, ^195^ ^]^ The local temperature increase (*δT*_NP_) at the spherical metal surface is proportional to the absorbed light power (*Q*), which is the product of absorption cross‐section (*σ*_abs_) and the irradiance of the incident illumination (*I*) as shown in Equation (6). *δT*_NP_ is inversely related to particle radius (*R*) and the thermal conductivity (*k*_S_) of immediate environment accounting for heat losses to the ambient medium

(5) $$\delta T_{\mathrm{NP}}=\frac{Q}{4\pi k_{S}R}$$

(6) $$Q=\sigma_{\mathrm{abs}}I$$

Compared to CW illumination, the pulsed illumination may induce a new set of effects, such as further temperature confinement, shorter temperature and pressure variations and vibration modes. The initial temperature increase experienced by the nanoparticles is dependent on the pulse duration. In this case, the rise in local temperature can be quantified based on Equation (7), where *F* is the fluence of laser pulse; *V* is the volume of the plasmonic nanoparticle; and *σ*_abs_ is the absorption cross‐section. *c* is the specific heat capacity of the nanoparticle and *ρ* is its mass density, which are the intrinsic physical properties of the catalyst

(7) $$\delta T_{\mathrm{NP}}^{0}=\frac{\sigma_{\mathrm{abs}}F}{V\rho c}$$

The formula is limited to modest temperature increases so as to maintain a constant heat capacity. By contrast, with the pulse duration exceeding the duration of the transient regime of plasmonic nanoparticles, these steps will occur simultaneously. When using nanosecond pulses, the maximum local temperature increase will not reach its maximum limit$\;\delta T_{\mathrm{NP}}^{0}\;$and the heating duration will be the same as the pulse duration.^[^ ^196^, ^197^, ^198^ ^]^

**Figure 13:**
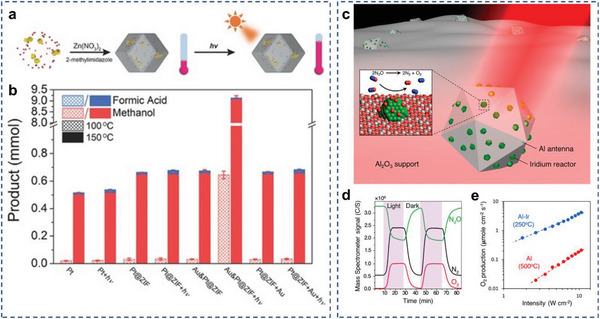
Photothermal heating for AR systems. a) Schematic illustration of the formation and the temperature variation under light irradiation of Au‐Pt@ZIF. b) Comparison of product yields over different catalysts. Reproduced with permission.^[^ ^205^ ^]^ Copyright 2016, WILEY–VCH. c) Schematic demonstration of the Al–Ir AR photocatalyst. d) Reactivity of the Al–Ir AR photocatalyst showing photoresponsive decomposition of N_2_O into N_2_ and O_2_ upon illumination. e) Arrhenius plot of N_2_O decomposition and apparent activation energy in the dark and under illumination. Reproduced with permission.^[^ ^206^ ^]^ Copyright 2019, American Chemical Society.

Heating through the photothermal effects of catalysts is typically highly localized, which can effectively elevate the local temperature at the catalyst‐reactant interfaces.^[^ ^16^, ^46^, ^159^, ^199^, ^200^, ^201^ ^]^ This can lead to a series of consequences, including an increase in the entropy of chemical reactions, modulation of the thermodynamics and kinetics of hot‐electron transfer, and increase in mass transfer.^[^ ^20^, ^189^, ^202^, ^203^, ^204^ ^]^ Photo‐induced heat release may sustain several pathways for stimulating the surface reaction, including 1) increasing the local thermodynamic temperature within the vicinity of the plasmonic nanoparticle, shifting chemical equilibria for endothermic processes forward toward a higher yield; 2) enhancing the adsorption and desorption processes of the surface chemistry. Zhan et al. found the nanoconfined thermal field in AR photocatalysts could increase the distribution of activated reactant to overcome the energy barriers and accelerate product removal.^[^ ^176^ ^]^ Zeng and coworkers demonstrated that through encapsulating Pt nanocubes and Au nanocages into a zeolitic imidazolate framework (ZIF‐8) to form a hybrid structure (Au‐Pt@ZIF) and by applying localized heating under Xe lamp light irradiation, the reaction temperature of CO_2_ hydrogenation can be effectively reduced (**Figure** **13**a,b).^[^ ^205^ ^]^ Specifically, Pt nanocubes served as a catalyst toward CO_2_ hydrogenation and Au nanocages with strong extinction could efficiently transform light to thermal energy. Notably, MOFs function as “heat insulators” to prevent heat from dispersing in solution. Under light irradiation at 150 °C, the turnover frequency (TOF) number of Au‐Pt@ZIF reached 1522 h^−1^, 13 times higher than that in the dark. For additional reaction types than CO_2_ reduction, Swearer et al. reported highly efficient decomposition of nitrous oxide (N_2_O) on Al–Ir AR plasmonic photocatalysts upon illumination from a band‐pass‐filtered supercontinuum picosecond pulsed laser source at 525 nm and 12 W cm^−2^ with 300 °C external heating (**Figure** 13c–e).^[^ ^206^ ^]^ As no appreciable change to the apparent activation energy was observed under illumination, the primary reaction enhancement mechanism for Al–Ir was likely photothermal heating rather than plasmon‐induced hot‐carrier contributions.

### 3.4 Synergistic Plasmonic Photocatalysis

Various plasmon‐associated pathways could be utilized synergistically to further enhance the catalytic efficacy. A most feasible approach is to concurrently induce and utilize the plasmonic photochemical and photothermal effect toward catalysis.^[^ ^159^, ^176^, ^185^, ^191^ ^]^ After hot electrons have been excited under light irradiation in plasmonic metals, the high‐energy electrons can relax to the ground state through nonradiative decay pathways. The photoinduced hot carriers can either interact with molecules adsorbed on the surface of the catalysts, or they can dissipate their energy into the environment to increase the local temperature.

**Figure 14:**
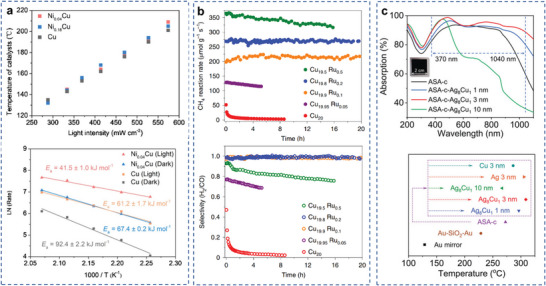
Synergistic plasmonic photocatalysis for AR systems. a) Dependence of surface temperature and rhenius plots for H_2_ production rate over pure Cu and Ni–Cu catalysts. Reproduced with permission.^[^ ^207^ ^]^ Copyright 2020, Elsevier B.V. b) Photocatalytic DRM performance under 19.2 W cm^−2^ white light illumination as a function of Ru concentration of the photocatalyst nanoparticles. Reproduced with permission.^[^ ^75^ ^]^ Copyright 2020, Springer Nature. c) UV–visible–near‐infrared absorption spectra and surface temperatures of Au based catalysts. Reproduced with permission.^[^ ^112^ ^]^ Copyright 2022, Wiley‐VCH GmbH.

Recently, several studies have demonstrated that by the rational design of plasmonic AR catalysts, the hot carriers and photothermal heating can contribute to the reaction simultaneously, resulting in not only enhanced activity but also modulated selectivity. As an example, Luo et al. used Cu–Ni AR photocatalysts for the high‐yield and selective production of H_2_ and acetaldehyde in solar‐driven ethanol dehydrogenation process. Mechanistic investigations revealed that the synergistic effects of hot carriers and photothermal heating over Cu–Ni AR photocatalysts played critical roles for the high activity. Hot electrons generated from Cu nanoparticles could migrate to Ni atoms, which simultaneously favored the separation of charge carriers and the activation of adsorbates (**Figure** **14**a).^[^ ^207^ ^]^ This work provided a promising pathway toward the utilization of solar energy and the construction of efficient plasmonic photocatalysts. Huang and coworkers presented a numerical study on the optimal design of the Ag–Pt core–shell nanostructures. Ag–Pt is demonstrated to be a good AR configuration because Ag has strong intrinsic plasmonic responses and a low imaginary part of dielectric function (*ε*_i_) in the visible region, while Pt is catalytically active and has a large *ε*_i_. Considering the hot carrier generation and transfer processes in both plasmonic photocatalysis and photothermal catalysis, the catalytically active sites at the Pt shell could be revealed by high local heating power densities.^[^ ^208^ ^]^

Resonant field‐enhancement mechanism has also been combined with plasmonic hot carrier‐induced reactivity in some pioneering works. Recently, Halas and coworkers designed an AR catalyst consisting of a Cu nanoparticle antenna with single‐Ru atomic reactor sites for efficient plasmonic DRM reaction.^[^ ^75^ ^]^ Under laser illumination, the catalyst achieved a turnover frequency of 34 mol H_2_ (mol Ru)^−1^ s^−1^ and photocatalytic stability over 50 h (**Figure** 14b). The isolated Ru atoms on the Cu surface offered high catalytic activity, while the plasmonic Cu antennas provided strong light absorption and thus efficient generation of hot carriers under illumination, which was the predominant mechanism in photocatalytic DRM. Hot carriers enhanced the rate of C—H activation and desorption of H_2_ at the Ru site, thereby inhibiting the rate of RWGS and coking, leading to high stability with high selectivity. This work provides a viable strategy for light‐driven low‐temperature methane reforming reactions.

Shao et al. designed a broadband plasmonic catalyst composed of Au‐based stacked plasmonic metamaterial as light absorber and Cu alloy as catalytic sites for efficient catalytic CO_2_ hydrogenation.^[^ ^112^ ^]^ The designed material realized ultra‐broadband light absorption (370–1040 nm) with more than 90% absorbance, enabling a high surface temperature over 300 °C and strong localized EM field. High performance of CO_2_ hydrogenation was achieved with a CO production rate of 1106 mmol m^−2^ and TOFs up to 1253 h^−1^. Additional catalytic pathways could also play a role, including photothermal heating and generation of charge carriers (Figure 14c). In situ high‐resolution XPS spectroscopy characterized the transfer path of hot carriers (from Au to Cu alloy active sites). Operando SERS spectroscopy and theoretical calculation gave insights on the reaction pathway. In short, the multieffect coupling in plasmonic AR photocatalysts will provide a strategy to achieve high catalytic performance under light illumination in different chemical reactions.

## 4 Deconvolution of Distinct Mechanisms in Synergistic Catalysis

While there is a consensus that all these plasmonic photocatalytic effects can enhance reaction rates, the relative contribution of each specific plasmonic catalytic mechanism remains less well understood, as identification and quantification of individual plasmonic pathway is challenging. In this section, several experimental and computational methodologies that have been utilized to distinguish between plasmonic photochemical and photothermal effects in plasmonic AR systems are summarized.

### 4.1 Light Intensity and Wavelength‐Dependence Study

The rate of chemical transformation is proportional to the rate of incident photons and therefore to the incident light power; while for high‐power or fs‐pulsed laser illumination, a super‐linear dependence is more common due to multiphoton absorption.^[^ ^195^, ^209^ ^]^ Thus, a linear dependence of the photocatalytic reaction rate on light intensity would typically indicate a photochemical process, whereas an exponential increase would rather be the signature of thermally driven transformations.^[^ ^168^, ^210^ ^]^

Varying the light beam diameter can also provide valuable information.^[^ ^211^, ^212^, ^213^, ^214^ ^]^ Under constant irradiance conditions, both hot‐carriers and thermal contributions will present a proportional dependence of reaction rate on the area of light beam. But under the constant‐power conditions, the rate of hot‐electron‐driven process is independent of the beam diameter, while thermal‐driven reactions show an inverse relationship between reaction rate and beam radius.

In addition, irradiation on versus off resonance can provide insights into the specific catalytic mechanism and potentially differentiate plasmonic photochemical processes from photothermal. For instance, by means of selective activation of distinct plasmon resonance modes, resonant energy transfer mechanism and photothermal/hot carrier mechanisms could be differentiated, as the former typically requires resonant oscillation frequencies, whereas the latter are usually connected with large optical cross‐sections but not necessarily spectral overlap.^[^ ^40^, ^127^, ^186^, ^187^ ^]^

### 4.2 Direct Temperature Measurements

The simplest method for determining potential thermal contributions is via direct measurement of the catalyst temperature under operando conditions. Infrared cameras have been used in experiments where a significant temperature increase is shown due to collective photothermal effects.^[^ ^86^, ^193^, ^215^, ^216^ ^]^ Alternatively, thermal measurements on macroscopic systems can be performed using thermocouples.^[^ ^29^, ^32^, ^217^ ^]^ However, these methods could only measure the macroscopic temperature. The local temperatures of the catalysts are often beyond their spatial resolutions. Other experimental approaches that have been used to measure temperatures at the nanoscale include scanning thermal microscopy,^[^ ^218^ ^]^ tip‐enhanced Raman spectroscopy,^[^ ^219^ ^]^ and computational methods.^[^ ^220^ ^]^ For instance, temperature could be calibrated based on the peak intensity of the anti‐Stokes or Stokes modes in Raman spectroscopy.

### 4.3 Activation Energy

To determine whether photon‐induced carriers contribute to the enhancement of the reaction rate, a straightforward approach is to compare the activation barriers under light and dark conditions at constant temperatures. The reduction in the value of activation barriers under light irradiation compared to dark conditions is indeed typical for a hot carrier‐driven mechanism.^[^ ^182^, ^221^, ^222^, ^223^ ^]^ However, as mentioned above, this method usually cannot be used to exclude the effect of plasmonic photothermal heating.

### 4.4 Time Scales

When illuminated, the change in temperature until reaching the steady state can have a kinetic time scale on the order of a few seconds to a few minutes in most cases, while the hot electron‐driven processes are often very fast, occurring at sub‐microsecond time scales due to the short lifetimes of the hot carriers and rapid relaxation processes. This fundamental difference in time scales can, in principle, be used to discriminate photothermal and photochemical effects.^[^ ^220^, ^224^, ^225^, ^226^, ^227^ ^]^ A slow increase in the chemical rate likely indicates a photothermal effect, whereas an instantaneous increase would rather suggest pure photochemical effects. For this kind of experiment, a precise estimation of the response time of the chemical sensor should be determined. However, this trend could be less evident for systems using plasmonic heterostructures with prolonged lifetimes of carriers, or that uses redox mediators to sustain subsequent catalytic cycles.^[^ ^228^, ^229^ ^]^

### 4.5 Kinetic Isotope Effects (KIE)

In isotope experiments, heavier isotopes require a higher energy input to reach the transition state. The KIE can be obtained by measuring the change in reaction rate after the introduction of labeled reactants. The electron‐driven process exhibits a larger KIE under light irradiation than thermally driven process under dark conditions, because the hot carrier injection could favor bond activation processes, facilitate chemical bond cleavage and thus the insertion of isotopes into the final products. By using a heavier isotope, the activation energy required for activating the chemical bonds increase, thus the difference in the capability of bond activation could be magnified.^[^ ^230^, ^231^, ^232^ ^]^

Meanwhile, other experimental approaches that have been used to demonstrate the existence of hot carrier in plasmon‐driven photocatalytic reactions include ultrafast spectroscopy,^[^ ^232^ ^]^ photoelectrochemical measurements,^[^ ^233^, ^234^ ^]^ photoluminescence,^[^ ^235^ ^]^ photocurrent, and selectivity studies.

## 5 Summary and Outlook

In summary, the direct coupling of plasmonic nanoantennas with catalytic nanoparticles into AR complex provides intriguing opportunities to construct efficient photocatalytic systems that combine the optical response and the intrinsic catalytic reactivity of the individual components. The favorable electronic structure of AR complexes and the signature LSPR effect enables various unique mechanisms in CO_2_ photocatalysis, including plasmon‐induced resonant energy transfer, charge carrier‐mediated photochemistry, forced plasmon, and photothermal effect. Given the great potentials of plasmon‐associated photophysical processes, there is no doubt that AR complexes hold great promise for applications in CO_2_ photocatalysis.

Further development of plasmonic AR‐based photocatalysts necessitates the successful solution of a series of challenges. Firstly, it is essential to deepen the fundamental understandings on the synergistic effect of plasmonic AR systems in CO_2_ photocatalysis. Particularly, the role of light or photochemistry in the catalytic process awaits further clarification. It is necessary to clearly identify and quantify the main mechanisms behind a plasmonic photocatalytic reaction.

One crucial factor that limits the deconvolution of photochemical and photothermal contributions is the inability to precisely measure the surface temperature at the nanoscale for the AR systems at the catalytic interface. Although photochemical contributions are claimed to be the major cause of AR catalytic reactivity in many works, photothermal contributions cannot be entirely excluded. This is because the hot carriers can thermalize within a few ps in a highly localized manner, and yet most macroscale temperature measurement techniques do not possess the adequate temporal and spatial resolution to capture the heat release. Calculation methods can predict the temperature increase on the surface of individual particles, but seem less relevant in the case of plasmonic coupling and heat accumulation in particle aggregates. In situ and operando characterization techniques need to be employed to obtain in‐depth insights on the catalytic mechanism, kinetics, and pathway; and thermal‐sensitive spectroscopic measurement could be combined with additional approaches for predicting equilibrium temperature^[^ ^29^ ^]^ in the catalytic conditions.

Last but not least, it remains a crucial bottle‐neck challenge to develop cost‐efficient AR photocatalysts that simultaneously combine efficient and broadband light harvesting capabilities, desirable catalytic reactivity, and stability. One crucial factor for this is through obtaining insight on the general design principles of plasmonic AR systems. Non‐noble metal‐based plasmonic metals (e.g., Al and Cu) and plasmonic semiconductor nanocrystals are likely to significantly reduce the cost of the plasmonic antenna,^[^ ^171^, ^236^, ^237^, ^238^ ^]^ although focused effort is needed to enhance their carrier density to comparable levels as conventional noble metals. Besides, more complex antenna designs are likely to further extend the light absorption range to the entire solar spectrum, and to augment the photo‐to‐chemical energy conversion efficiency. Plasmonic superstructures and plasmonic alloys architectures could represent a few feasible options along this path.

With the advances in materials preparations, in situ characterizations and theoretical calculations, the field of AR‐based photocatalytic CO_2_ conversions has a bright future. AR not only creates a unique set of conditions with great significance from the points of view of fundamental research to chemical reactions, but also provides a promising approach to facilitate light‐driven chemical conversions for effective utilization of solar energy. We hope this review will bring together researchers with different backgrounds to take adventure in this emerging and exciting area.

## Conflict of Interest

The authors declare no conflict of interest.
